# Copine-5-IgG-related autoimmune encephalitis: a novel paraneoplastic neurological syndrome with a strong melanoma association

**DOI:** 10.1186/s12974-026-03793-4

**Published:** 2026-07-27

**Authors:** Matthias Elstner, Sven Jarius, Brigitte Wildemann, Katharina Drüschler, Kathrin Borowski, Bianca Teegen, Corinna Bien, Janek Nagel, Ilka Kleffner, Markus Höltje, Jürgen Haas, Klemens Ruprecht, Yvonne Müller, Christiane Radzimski, Lars Komorowski, Ramona Miske, Madeleine Scharf

**Affiliations:** 1 Department of Neurology, University Hospital Rechts der Isar, Technical University Munich, Munich, Germany; 2 Rehaklinik Tschugg, Tschugg, Switzerland; 3 Division of Neuroimmunology, Department of Neurology, University of Heidelberg, Heidelberg, Germany; 4 Department of Neurology, University of Heidelberg, Heidelberg, Germany; 5 Clinical Immunological Laboratory Prof. h.c. (RCH) Dr. med. Winfried Stöcker, Groß Grönau, Germany; 6 Laboratory Krone, Bad Salzuflen, Germany; 7 Department of Neurology, University of Bochum, Knappschaftskrankenhaus, Bochum, Germany; 8 Institute for Integrative Neuroanatomy, Charité – Universitätsmedizin Berlin, Corporate Member of Freie Universität Berlin and Humboldt-Universität zu Berlin, Berlin, Germany; 9 Institute for Experimental Immunology, EUROIMMUN Medizinische Labordiagnostika AG, Seekamp 31, Luebeck, 23560 Germany

**Keywords:** Copine-5 (CPNE5), Autoantibodies, Autoimmune encephalitis, Paraneoplastic neurological syndrome, Melanoma, Immunoglobulin G (IgG), Limbic encephalitis, Striatal encephalitis, Brainstem encephalitis, Cognitive decline, Seizures, Epilepsy, Secondary parkinsonism, Hypoactive delirium, Abulia, Mutism, Basal ganglia, Caudate nucleus, Deep gray matter, Cerebellum, Temporal lobe, Hippocampus

## Abstract

**Background:**

Although significant progress has been made in identifying antigen targets in autoimmune encephalitis (AIE), in a substantial proportion of patients with presumed AIE no known autoantibodies can be detected. Herein, we describe a novel autoantibody reactivity, directed against copine-5 (CPNE5), in patients with AIE and melanoma.

**Methods:**

Patients were identified through routine clinical testing for anti-neural autoantibodies by indirect immunofluorescence on neuronal tissue sections. The antigen was identified by immunoprecipitation and mass spectrometry and confirmed by recombinant protein assays.

**Results:**

Serum and cerebrospinal fluid (CSF) immunoglobulin G (IgG) from four patients was found to exhibit distinct binding to cerebellar and hippocampal neurons. Copine-5 was identified as the target antigen. Clinical manifestations included cognitive decline, confusion/disorientation, psychosis, seizures and signs and symptoms compatible with basal ganglia involvement (movement disorders, including secondary parkinsonism, and/or abulia/akinetic mutism), alongside pain (including painful tonic spasms and focal allodynia), dysarthria, dysphagia, and abducens nerve palsy. Brain magnetic resonance imaging disclosed hyperintense lesions in the basal ganglia and the temporal lobe. CSF analysis revealed mild pleocytosis, intrathecal IgG synthesis, and blood/CSF barrier dysfunction. Of particular note, all four patients had melanoma, with an occult primary in three. In one patient, melanoma diagnosis and immunotherapy with the anti-PD-1 immune checkpoint inhibitor nivolumab preceded the onset of AIE by 9 months. Glucocorticoid treatment and/or intravenous immunoglobulins led to transient improvement in at least three patients; however, all relapsed and three progressed to hypoactive delirium or mutism. Plasma exchange and cyclophosphamide treatment were followed by clinical improvement and stabilization in one patient, but potentially contributed to fatal infectious complications. Copine-5-IgG belonged to the strongly complement-activating IgG1 subclass and was produced intrathecally. Serum titers ranged between 1:1000 and 1:100,000.

**Conclusions:**

Copine-5 is a novel autoantibody target in AIE. Testing for anti-copine-5 autoantibodies should be included in the diagnostic workup of patients with AIE, especially, but not exclusively, if associated with melanoma or positive melanoma-associated tumor markers. Further studies investigating the immunopathogenesis of copine-5-related autoimmunity and the potential significance of anti-copine-5 as a novel paraneoplastic serological marker and of copine-5 as a histopathological tumor marker in patients with suspected melanoma are highly warranted.

**Supplementary Information:**

The online version contains supplementary material available at 10.1186/s12974-026-03793-4.

## Introduction

Autoimmune encephalitis (AIE) is characterized by immune-mediated brain inflammation that leads to neuronal dysfunction, clinically manifesting with a range of neurological and psychiatric symptoms. In many patients, AIE is associated with the presence of serum and/or cerebrospinal fluid autoantibodies that target neuronal antigens within the central nervous system (CNS), resulting in variable pathophysiological downstream effects depending on the antigen [1–3]. AIE often occurs in a paraneoplastic context, manifesting as limbic encephalitis, cerebellar ataxia (also known as cerebellar degeneration), encephalomyelitis, or myelopathy. Other known manifestations include stiff person syndrome, Lambert-Eaton myasthenic syndrome, myasthenia gravis, opsoclonus myoclonus syndrome, and neuromyotonia (Isaac’s syndrome). Paraneoplastic neurological syndromes (PNS) may also affect the peripheral nervous system, causing peripheral neuropathy, and the autonomic nervous system, causing autoimmune dysautonomia. Many PNS are strongly associated with certain tumor entities, such as anti-Hu syndrome, which predominantly occurs in patients with small cell lung cancer, or anti-Yo syndrome, which mainly occurs in patients with ovarian cancer.

Testing for anti-neural autoantibodies has clinically important diagnostic, therapeutic, and prognostic implications. By providing strong evidence for an autoimmune etiology in patients presenting with encephalitis of unknown cause, the demonstration of anti-neural antibodies can guide prognostically important treatment decisions. Obligatory or facultatively paraneoplastic anti-neural autoantibodies often help to identify underlying tumors, which are frequently occult at the time of neurological onset. In recent years, significant progress has been achieved in discovering new anti-neural autoantibodies [1–3]. However, in many patients with suspected AIE no anti-neural antibodies have yet been identified. In these seronegative cases, the diagnosis of AIE is still possible according to current diagnostic criteria, based on clinical, radiological, and cerebrospinal fluid (CSF) findings suggestive of AIE, but the risk of misdiagnosis exists [4, 5].

Here, we describe copine-5 as a novel antibody target in AIE characterized by predominantly basal ganglia and neuropsychiatric symptoms. In all four cases, the presence of copine-5 immunoglobulin G (IgG) was associated with melanoma, which potentially renders the syndrome a novel PNS.

## Patients and methods

### Patients

All four copine-5-IgG-positive patients (3 × male, 1 × female) were treated at the contributing hospitals for suspected AIE. Samples of serum and, if available, CSF were tested for anti-neural antibodies in all cases as part of the patients’ routine diagnostic workup. Written informed consent for publication was obtained from the three index patients or their legal caregivers. Subsequently, a fourth copine-5 antibody-positive patient was identified; however, the patient was lost to follow-up and only basic data are therefore presented. In addition, serum samples of 50 anonymized healthy blood donors (HD) were analyzed.

### Indirect immunofluorescence assay with brain tissue and recombinant HEK293 cells

Indirect immunofluorescence assay (IIFA) using microscopy slides with a biochip array of rat brain cryosections combined with recombinant human embryonic kidney (HEK293) cells separately expressing 35 different brain antigens (Hu, Yo, Ri, CV2, PNMA2, ITPR1/Sj [37], Homer 3, CARP VIII, ARHGAP26/Ca [36], ZIC4, DNER/Tr, GAD65, recoverin, GABA_B receptor, glycine receptor, DPPX, IgLON5, glutamate receptors [NMDA, AMPA, mGluR1, mGluR5, GLUR-delta2], LGI1, CASPR2, AQP4, MOG, ATP1A3, NCDN, flotillin1/2, KCNA2, AP3B2, SEZ6L2, CNTN1, NF155, and NF186) was performed as described previously [6]. Additionally, IIFA was performed using recombinant acetone-fixed HEK293 cells expressing copine-5 and empty vector-transfected HEK293 cells as control substrate (RC-IIFA). End titers of positive samples were determined using a dilution scheme based on the square root of 10 (1:10, 1:32, 1:100, 1:320, etc.). Bound patient IgG was detected by use of a goat anti-human IgG antibody labeled with FITC (Euroimmun) or Alexa Fluor^®^ 488 (Jackson ImmunoResearch, Ely, UK), as described previously. Empty vector-transfected HEK293 cells were used as control substrates. Cell nuclei were counterstained with TO-PRO-3 Stain (ThermoFisher Scientific, Darmstadt, Germany). For competitive immunofluorescence assay, 1:100 diluted patient serum was preincubated with 1:2 diluted extract either from copine-5-expressing HEK293 cells or from empty vector-transfected control cells 1 h prior to incubation on tissue IFA slides.

### Immunoprecipitation and mass spectrometry

Immunoprecipitation (IP) was performed with 30 µl serum and 200 µl rat brain tissue homogenate in 500 µl solubilization buffer [6]. The supernatants were incubated for 3 h with Protein G Dynabeads (ThermoFisher Scientific). Beads were eluted with NuPAGE LDS sample buffer (ThermoFisher Scientific) containing 25 mmol/l dithiothreitol followed by sodium dodecyl sulfate polyacrylamide gel electrophoresis (SDS-PAGE, NuPAGE; ThermoFisher Scientific) and Coomassie Brilliant Blue (G-250) (Merck) gel staining. Selected protein bands were analyzed by mass spectrometry as described elsewhere [2]. Eluate fractions were used for immunoblot analyses.

### Immunoblotting

Immunoprecipitated cerebellum lysate fractions were subjected to immunoblotting as previously described [7]. Membranes were incubated for 3 h with rabbit anti-copine-5 antibody (1:500, Sigma Aldrich HPA031369) in 1:5 diluted sample buffer (Euroimmun), followed by incubation with anti-rabbit IgG-AP (1:2000, Jackson ImmunoResearch).

### Recombinant expression of full-length copine-5 in HEK293 cells

The cDNA for isoform 2, encoding a partial human copine-5 sequence, was obtained from Source BioScience UK Limited as clone IRATp970E0779D. The cDNA sequence encoding amino acids 1-292, which are absent in isoform 2, was generated by a gene synthesis service (MWG-Biotech, Ebersberg, Germany) and integrated into the cDNA of isoform 2 to generate the full-length canonical isoform 1 (Q9HCH3-1). The coding sequences were amplified by PCR using the template cDNA and DNA oligonucleotide primers. The amplification products were digested and ligated with linearized pTriEx-1 (Merck, Darmstadt, Germany). For all RC-IIFA and competitive IIFA experiments the canonical full-length sequence of copine-5 was used.

### Preparation of cerebellar cultures

Cerebellar neurons were prepared at embryonic day 19 from SWISS mice. Brains were collected in ice-cold PBS supplemented with 0.6% glucose. After removal of the meninges, the cerebella were separated from the whole brains. The cerebella were then transferred into neurobasal medium (NBM) containing 2% B27, 1mM L-glutamine, and 1% Pen/Strep. Next, tissue was homogenized for cell dissociation using a glass Pasteur pipette (cell suspension was passed 8× through the pipette). Cells were centrifuged for 2 min at 800 rpm. The pellet was then resuspended in NBM-starter medium (NBM + 0.25 mM sodium glutamate) and dissociated again 6× using fine plastic pipettes. Cells were again spun down at 800 rpm for 2 min. Cells were counted and seeded in NBM-starter medium additionally supplemented with 10% FCS at a density of 3–5 × 10^6^ per ml in 10 µl droplets using glass coverslips in 24-well plates precoated with poly-L-lysine at 500 µg/ml. Neurons were allowed to attach to the coverslips by incubation in droplets for 3 h at 5% CO_2_. Thereafter, NBM-starter medium was added to 500 µl with 1% FCS added. Neurons were cultured for 3 days before the medium was exchanged for DMEM/F12 Ham’s medium supplemented with 1% FCS, 100 µM putrescine, 30 nM sodium selenite, 1.4 mM L-glutamine, 40 nM progesterone, 20 µg/ml insulin, 0.5 ng/ml triiodothyronine (T3), and 200 µg/ml transferrin. Neurons were allowed to grow until day 14 in vitro before immunostaining. On day 8 in culture, 50% of the medium was replaced with fresh culture medium supplemented with bovine serum albumin (BSA) at 100 µg/ml.

### Staining of Purkinje cells

Medium was removed, and cells were washed with PBS. For some experiments, cells were fixed using 4% paraformaldehyde in PBS for 20 min at room temperature and subsequently incubated with blocking solution (0.1% Triton; 5% NGS; 2% BSA; PBS) for 1 h at room temperature. To identify Purkinje cells, cultures were stained against calbindin using a polyclonal rabbit anti-calbindin D-28k antibody obtained from Swant (#CB300PUR; Burgdorf, Switzerland) for 24 h at 4 °C together with patient sera or CSF at given dilutions. Cells were then washed with PBS and incubated with secondary antibodies – Alexa Fluor 488-conjugated goat-anti-human IgG (Biozol, Hamburg, Germany, #109-545-003) and Alexa Fluor 594-conjugated goat-anti-rabbit (Thermo Fisher Scientific, Waltham, MA, USA, #A11037) – in secondary antibody solution (2% BSA in PBS) for 90 min at room temperature. Cells were again washed with PBS, stained with DAPI (4‘, 6-diamidino-2-phenylindole) for 10 min, and mounted with Immu-Mount (Thermo Fisher Scientific). For live staining, cerebellar cultures were incubated with patient CSF for 24 h prior to fixation.

### Immunoglobulin G subclass analysis

For determination of immunoglobulin G (IgG) subclasses, the FITC-labeled goat anti-human IgG secondary antibody used in the TBA experiments was replaced by sheep anti-human IgG1-, IgG2-, IgG3-, and IgG4-specific secondary antibodies (The Binding Site, Schwetzingen, Germany) and an AF568-labeled donkey anti-sheep IgG (Invitrogen).

### Antibody specificity index

Quantification of intrathecal antibody production was based on quotients of CSF to serum for both copine-5-specific IgG and total IgG: QIgG [spec] = IgGspec (CSF)/IgGspec (serum), and QIgG [total] = IgGtotal (CSF)/IgGtotal (serum). Antibody titers were measured semi-quantitatively by indirect immunofluorescence on cerebellar sections as described above [8]. Total IgG and albumin concentrations in paired serum and CSF were determined by nephelometry (BN ProSpec, Dade Behring, Germany). Intrathecal synthesis of anti-copine-5 antibodies was inferred from the antibody index (AI), calculated as follows: AI = QIgG [spec]/QIgG [total] when QIgG [total] < Qlim, and AI = QIgG [spec]/Qlim when QIgG [total] ≥ Qlim [8]. The upper reference limit for QIgG [total] (Qlim) was derived using Reiber’s formula to correct for potential underestimation of intrathecal antigen-specific synthesis due to blood–CSF barrier dysfunction. AI values > 4 were taken as evidence of intrathecal production of anti-copine-5 IgG [9].

## Results

### Clinical and paraclinical features

Clinical and paraclinical data were available for analysis from three patients and are summarized in Table 1. Disease onset was subacute in all three patients, followed by progressive deterioration.

**Table 1:** Demographic, clinical, and paraclinical features as well as data on treatment and outcome of three patients with copine-5-IgG-associated autoimmune encephalitis. All patients tested negative for antibodies against Hu, Yo, Ri, CV2, ANNA-3, Tr/DNER, Ma1, Ma2/Ta, GAD65, NMDAR, AMPAR, LGI1, CASPR2, Zic4, DPPX, CARP VIII, GlyR, mGluR1, mGluR5, GABA_A-R, GABA_B-R, ARHGAP26/anti-Ca, ITPR1/anti-Sj, Homer 3, AQP4, MOG, myelin, recoverin, neurochondrin, neurexin-3alpha, GluR-delta2, flotillin1/2, IgLON5, SEZ6L2, AP3B2, contactin-1, NF155, NF186, AT1A3, and KCNA2

Sex, age	Patient 1	Patient 2	Patient 3
	Female, 64	Male, 70	Male, 75
Clinical diagnosis	Paraneoplastic AIE (LE with seizures)	Paraneoplastic AIE (LE with basal ganglia involvement)	Paraneoplastic AIE (LE, BSTE)
Clinical features	One-week prodromal phase with fatigue, loss of appetite/indifference to hunger with marked weight loss, depressed mood, double vision (N. VI paresis); seizures (generalized tonic-clonic, dyscognitive), abulia, progressing to hypoactive delirium	Subacute cognitive dysfunction, memory dysfunction, mild dysarthria; rigidity of right extremities, postural instability, tremor right hand, micrographia, distal paresis of right leg; deterioration after discontinued targeted therapy: possibly seizures; painful tonic spasms of the right extremities; focal pain and allodynia; hypophonia; increasing impairment of verbal memory and verbal fluency; last follow-up: bedridden, reduced motivation/initiation/spontaneous activity, hypokinetic mutism	Initial presentations: dysarthria, dysphagia; later fluctuating confusion; disoriented; affective incontinence, aggression, hallucinations, delusional symptoms; psychosis not responsive to neuroleptic treatment; sleep-wake cycle disturbance; altered level of consciousness/somnolence, occasional attempts at dysarthric speech production, hypoactive delirium; no paresis
MRI findings	Initial MRI and follow-up MRI 2 weeks later: Multiple T2 FLAIR hyperintense, contrast-enhanced brain lesions (subcortical, paraventricular, hippocampal, internal capsule, basal ganglia)	Prominently left-sided T2/FLAIR hyperintensities extending from basal ganglia to temporal lobe, minimal Gd enhancement; follow-up: brain atrophy with dilatation of the lateral ventricles	CT/MRI: Lesions left putamen, lenticulostriate, insula; hippocampal remnant cysts, Gd MO+pons; global brain atrophy, inner/outer spaces↑; periventr. WM T2 lesions, DD: microangiopathic
EEG	Abnormal EEG (evidence of increased cerebral excitability: preserved α rhythm at 9–10 Hz with normal reactivity, but intermittent, irregular high-amplitude delta bursts [bilateral frontal regions]); no focal abnormalities	N.a.	Abnormal EEG consistent with mild, diffuse cerebral dysfunction (globally low voltage with poorly developed posterior dominant rhythm 8–9 Hz and markedly reduced reactivity); no focal abnormalities, no epileptiform activity or ictal evolution
Initial CSF	6 cells/µl, ly (no tumor cells), normal TP, glucose, L-lactate, and NSE; 14-3-3 neg	8 cells/µl (ly-mo; no tumor cells); Q_Alb_↑ (13.1); QIgG↑ (14.3); IgG-IF 25%; normal QIgA/M; TP↑ (95 mg/dl); L-lactate normal	24 cells/µl, CSF TP↑ (106 mg/dl)
Follow-up CSF	2x normal WCC, ly-mo (no tumor cells), normal TP, L-lactate, glucose, QAlb↑, type 3 OCB, normal QIgG	2× normal WCC (ly-mo), no tumor cells; QAlb 1 × 18.5, 1 × 10.7; 1× type 2 OCB, 1× type 3 OCB, 2× normal QIgG/A/M	7/7/3 cells/µl (monon > pm), no tumor cells (morphol., S100); QAlb↑ (12/11/17); 2×OCB pos; 3×QIgG/A/M normal; CSF TP↑ (55/51/79 mg/dl), 3×L-lactate and glucose levels normal
Ab findings	Copine-5 CBA: 1:32,000 (serum), CSF not tested	Copine-5 CBA: 1:100,000 (serum), 1:10,000 (CSF)	Copine-5 CBA: 1:1000 (serum), 1:320 (CSF)
Microbiology	Neg: HSV1/2, VZV, *BB*, HIV, TBE, HepA/B/C	Neg: HSV-1-, HSV-2-, and VZV-PCR, serology for BB, HepA/B/C	Neg: HSV1/2, VZV, CMV, HHV6, enterovirus, CC, NM
Tumor association	Amelanotic melanoma, stage III (cTX, cN1, pMX; axillary LN biopsy, occult primary); AE1/AE3-neg, CK7-neg, S100-pos; BRAF mutation-pos [V600E])	Melanoma (pulmonary mass biopsy, occult primary; cTX, cNX, pM1b, stage IV; SOX-10+++, S100+; mutational state: BRAF p. Val600Glu; TERT promoter C250T; ERBB3 p.Leu97fs*26)	Ulcerated plantar melanoma (pT3b, N3c, cM1b, AJCC stage IV; BRAF-mutation: wt)
CT/PET-CT	Whole-body PET-CT: hypermetabolism LN (left axilla)	Spiculated mass left lung apex with broad-based pleural involvement, LN metastases at the aortic arch	Thoracic/abdominal CT: no lesions suspicious for metastases
Systemic complications	Splenic infarction, thrombosis abdominal aorta	Mild anemia	Aspiration pneumonia due to dysphagia
Tumor treatment	Vemurafenib, cobimetinib	Encorafenib and binimetinib (discontinued due to dermatological side effects)	Nivolumab, ipilimumab
Immunotherapy	Prednisolone 1 mg/kg (improvement; mRS 2→1); IVMP 1 g/day for 5 days at first seizure (mRS 5→5); 5 cycles of PEX (improvement) and 3 cycles of CYP (stabilization; mRS 5→3)	IVMP 1 g/d for 3 days (transient improvement); no further IM/IS treatment documented; pregabalin and amitriptyline; mRS at last follow-up: 4	IVMP and 3d IVIG (slight transient improvement); repeat IVMP (no significant improvement)
Outcome	SIRS following repeated UTI, died 7 mo after neurological onset and 5 mo after start of CYP	Alive 17 mo after neurological onset, mRS 4	Alive 15 mo after neurological onset, nursing home

*Abbreviations*: *AB* Antibody, *AIE  *Autoimmune encephalitis, *AMPAR * α-Amino-3-hydroxy-5-methyl-4-isoxazolepropionic acid receptor, *ANNA-3 * Anti-neuronal nuclear antibody type 3, *AP3B2 * Adaptor-related protein complex 3, beta 2 subunit, *AQP4 * Aquaporin-4, *ARHGAP26*  Rho GTPase-activating protein 26 (also known as anti-Ca), *AT1A3 * ATPase Na+/K+ transporting subunit alpha 3, *BB Borrelia burgdorferi, BG * Basal ganglia, *BRAF* v-Raf murine sarcoma viral oncogene homolog B1, *BSTE * Brainstem encephalitis, *CBA * Cell-based assay, *CC* Cryptococcus, *CK7 * Cytokeratin 7, *CSF* cerebrospinal fluid, *CYP *Cyclophosphamide, *CTL* Control, *DNER* Delta/Notch-like EGF-related receptor, *DPPX * Dipeptidyl-peptidase-like protein 6, *EEG * Electroencephalogram, *ERBB3 * Receptor tyrosine-protein kinase erbB-3, *ERC1* ELKS/RAB6-interacting/CAST family member 1, *FLAIR* Fluid-attenuated inversion recovery, *TBE * Tick-borne encephalitis, *GABA_A-R * Gamma-aminobutyric acid receptor type A, *GABA_B-R * Gamma-aminobutyric acid receptor type B, *GAD65 * Glutamic acid decarboxylase 65, *Gd* Gadolinium,  *GluR-delta2* Glutamate receptor delta-2 subunit, *GlyR* Glycine receptor, *pm * polymorphonuclear leukocytes, *HSV1/2 * Herpes simplex virus types 1 and 2, *IgG* Immunoglobulin G, *IgG-IF* Intrathecal IgG fraction, *IgLON5 * Immunoglobulin-like cell adhesion molecule 5, *IM/IS* Immunomodulatory/immunosuppressive, *ITPR1* Inositol 1,4,5-trisphosphate receptor type 1 (also known as anti-Sj), *IVIG * Intravenous immunoglobulin, *IVMP * Intravenous methylprednisolone, *KCNA2 * Potassium voltage-gated channel subfamily A member 2, *LGI1 * Leucine-rich glioma-inactivated 1, *LN * Lymph node, *LP * Lumbar puncture, *ly-mo * lympho-monocytic, *Ma1 * Ma1 onconeural antigen, *Ma2 * Ma2 onconeural antigen, *mGluR1 * metabotropic glutamate receptor 1, *mGluR5 * metabotropic glutamate receptor 5, *NM *Neisseria meningitidis*, MO * Medulla oblongata, *mo * month(s), *monon * mononuclear, *mRS * modified Rankin Scale, *MRI * magnetic resonance imaging, *MOG * Myelin oligodendrocyte glycoprotein, *MRZ * Measles, rubella, and zoster reaction, *N.a.* not available, *N. VI paresis * cranial nerve VI (abducens nerve) palsy, *NF155 * neurofascin 155, *NF186 * Neurofascin 186, *NMDAR N*-methyl-D-aspartate receptor, *OCB * Oligoclonal IgG bands, *PEX * Plasma exchange, *QAlb * Albumin CSF/serum ratio, *QIgG* IgG CSF/serum ratio, *Ri * Ri onconeural antibody, *SEZ6L2 * Seizure-related 6 homolog-like 2 protein, *SIRS * Systemic inflammatory response syndrome, *SOX-10 * SRY-related HMG-box gene 10, *Ta * onconeural antigen Ta (= Ma2), *TERT * Telomerase reverse transcriptase, *TNM * Tumor node metastasis staging system, *TP * Total protein, *VZV * Varicella-zoster virus, *WCC * White cell count, *WM * White matter, *wt * Wild type, *Yo * Yo onconeural antibody, *Zic4 * Zinc finger protein 4

#### Clinical presentation

Patient 1 presented with a 1-week history of fatigue, loss of appetite (weight loss of 7 kg), low mood, and 6th nerve palsy with double vision. Two weeks after onset, she experienced generalized tonic-clonic as well as dyscognitive seizures and, finally, progressed to abulia and hypoactive delirium. Patient 2 developed dysarthria early in the disease course, together with cognitive dysfunction, and went on to develop central paresis of the right leg and signs of secondary parkinsonism. Later, he suffered in addition from painful tonic spasms. Patient 3 initially presented with dysarthria and dysphagia and later developed confusion and somnolence. More detailed case reports can be found in the Appendix. Patient 4 presented with a movement disorder and polyneuropathy, but no further information is available.

#### Magnetic resonance imaging (MRI) findings

**Fig. 1:**
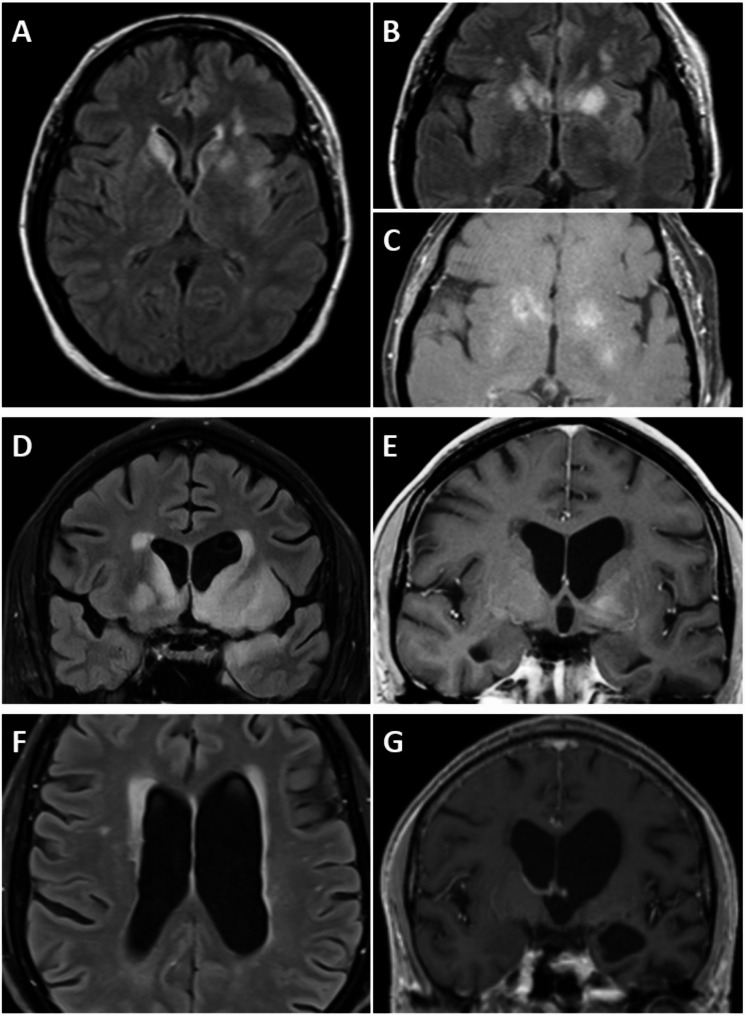
Magnetic resonance imaging (MRI) findings in two copine-5-IgG-positive patients. **A**-**C** MRI showing lesions in the basal ganglia (especially the heads of the caudate nuclei), paraventricular region, hippocampus, and frontal lobe in patient 1, two weeks after clinical onset, on axial (A) and coronal (**B**) T2/FLAIR imaging, with gadolinium (Gd) enhancement in the basal ganglia notable on coronal T1 imaging (**C**). **D**-**E** Coronal MRI imaging in patient 2 showing predominantly left-sided, extensive hyperintensities extending from the basal ganglia to the temporal lobe in T2 dark fluid imaging (**D**) with mild Gd enhancement in the basal ganglia (greater on the left than on the right) on T1 imaging 3 months after neurological onset (**E**). **F **(axial) and **G** (coronal): Periventricular T2 dark fluid hyperintensities and dilatation mainly of the left-sided lateral ventricle in patient 2 at month 6 (F; no coronal sequences available) and marked brain atrophy with dilatation of mainly the left lateral ventricle at month 21 on T1/Gd-weighted imaging (**G**)

In patients 1 and 2, in whom symptom progression was faster and copine-5-IgG titers were higher, initial MRI studies revealed T2/FLAIR-hyperintense lesions with only mild contrast enhancement in the basal ganglia (including the caudate nucleus) and the temporal lobe (Fig. 1). In patient 3, the radiological findings reportedly included a lesion in the left putamen, lenticulostriate and insular lesions, hippocampal changes considered to correspond to remnant cysts, and possible Gd enhancement in the medulla oblongata and pons. In addition, global brain atrophy with expansion of the inner and outer CSF spaces, both supra- and infratentorially, was noted, along with marked periventricular white matter T2 hyperintensities. However, a microangiopathic origin of some of the lesions in patient 3 was considered in the differential diagnosis.

#### CSF findings

CSF assessment initially revealed mild lymphomonocytic pleocytosis (6, 8, and 24 cells/µl, respectively) in all three cases with available data, which normalized upon corticosteroid therapy in two cases. Intrathecal total IgG synthesis was observed in patients 1, 2 and 3 (no data available for patients 4), as evidenced by an increased CSF/serum IgG ratio (QIgG) and/or CSF-restricted oligoclonal bands (OCB) (Table). Repeated CSF analyses of patient 2 showed normalization of the IgG ratio but persistent CSF-restricted OCB, suggesting reduced but ongoing intrathecal IgG synthesis. OCB remained detectable at follow-up also in patient 3 (no data available for patients 1 and 4). Blood–CSF barrier (BCB) dysfunction, as defined by an increased CSF/serum ratio (QAlb), was present at least in patient 1 (determined only at repeat CSF analysis), patient 2 (severely increased QAlb at first lumbar puncture [LP] and less severe but persistent BCB dysfunction at second and third LP) and patient 3 (at first and two follow-up LPs); no data on OCB, QIgG, and QAlb were available for patient 4.

#### Oncological findings

Patients 1, 2, and 4 were all diagnosed with (occult) melanoma upon tumor screening prompted by the presence of AIE. In patient 1, a suspicious lymph node was found on physical examination. Whole-body combined positron emission tomography and computed tomography (PET-CT) showed a solitary hypermetabolic lymph node lesion, but was otherwise unremarkable. Histopathological examination after needle biopsy revealed a grade III melanoma. In patient 2, thoracic CT demonstrated a spiculated mass in the left lung apex along with mediastinal lymph nodes suspicious for metastases at the aortic arch. Pulmonary nodule biopsy confirmed melanoma. In neither patient was cutaneous melanoma detected. By contrast, patient 3 had been diagnosed with a melanoma 9 months before onset of the neurological symptoms.

#### Treatment and clinical outcome

Due to the presence of BRAF mutations, patient 1 received combination therapy with vemurafenib (BRAF inhibitor) and cobimetinib (MEK inhibitor), and patient 2 was given a combination of the BRAF inhibitor encorafenib and the MEK inhibitor binimetinib. The melanoma of patient 3 had shown no BRAF mutation and had been treated with the checkpoint inhibitor ipilimumab and the PD-1 (programmed death 1) inhibitor nivolumab (last treatment 1 month before onset of AIE). 

Glucocorticoid treatment was followed by transient improvement in patients 1–3 (no data on patient 4). Secondary progression prompted escalation therapy with plasma exchange (patient 1) and IVIG (patient 3), resulting in modest clinical improvement in both patients. Patient 1 was subsequently started on long-term immunosuppressive therapy with cyclophosphamide (CYP), which was followed by neurological stabilization. However, the patient died 5 months after initiation of CYP and 7 months after neurological onset from infectious complications in a palliative setting.

### Serological findings

#### Indirect immunofluorescence

**Fig. 2:**
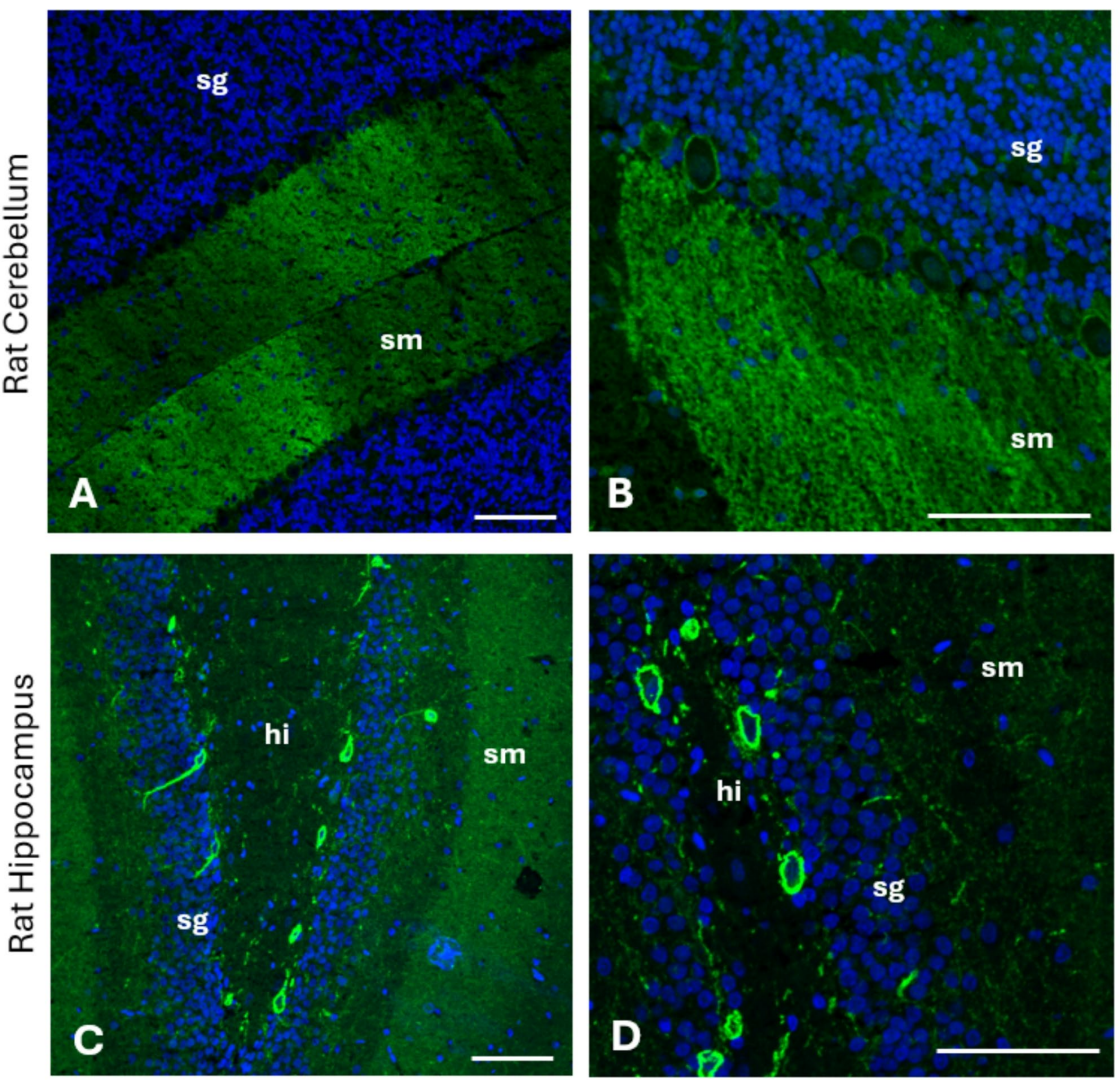
Binding of IgG from the index patient to rat brain tissue sections. **A **and **B** Binding of patient serum IgG to the molecular layer and Purkinje cell layer of rat cerebellum sections (B: higher magnification, scale bar: 100 μm). **C **and **D** Binding of patient serum IgG to the cell membrane of a subpopulation of hippocampal neurons, most of which are located directly below the granular layer (D: higher magnification, scale bar: 100 μm). Binding of patient IgG was visualized using an Alexa488-labeled, Fcγ-specific goat anti-human IgG secondary antibody (green). TO-PRO-3 stain was used to stain nuclei (blue). An identical IgG binding pattern was seen with CSF samples from patients 2 and 3 and serum samples from patients 2, 3, and 4 (not shown). Sg: stratum granulosum; sm: stratum moleculare; hi: hilus

**Fig. 3:**
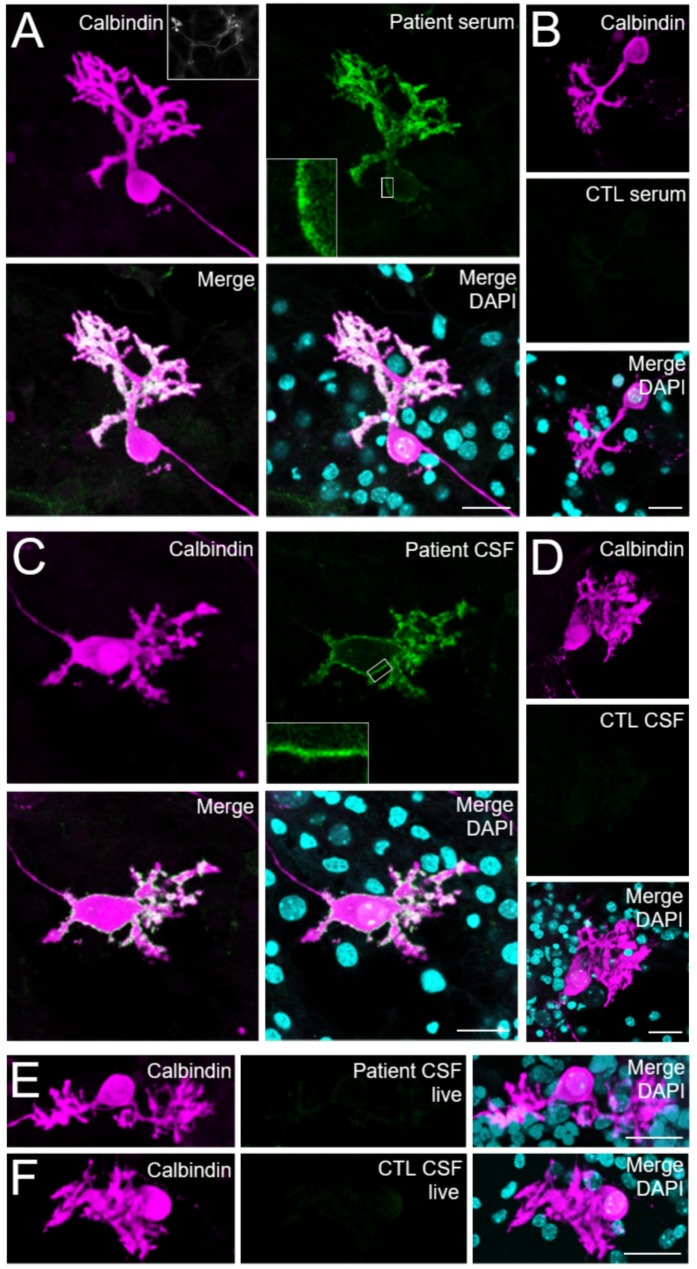
Staining of cultured Purkinje cells by patient IgG. Murine cerebellar cultures were prepared at embryonic day 19 and kept for 14 days in culture. After fixation, cells were incubated with a primary rabbit antibody against calbindin, used as a Purkinje cell marker, and serum and CSF, respectively, from patient 2 at a dilution of 1:1000. Bound rabbit and human IgG were detected by use of Alexa594-labeled (magenta) and Alexa488-labeled (green) detection antibodies, respectively. **A** Patient IgG was found to bind to the dendritic tree and soma of Purkinje cells in a membrane-accentuated fashion (see inset for morphology and details of somatic staining). **B** Incubation with serum from healthy human donors at the same dilution showed no specific staining of Purkinje cells. **C** Incubation of cerebellar cultures with the patient’s CSF at a dilution of 1:50 resulted in membrane-accentuated staining comparable to that observed with serum (see inset for detail). **D** Incubation with a control CSF sample yielded no staining. PC staining by patient IgG was only seen with fixed and permeabilized PCs (A-D) but not with live PCs (**E**-**F)**, in line with an intrathecal location of the antigen. DAPI (4′,6-diamidino-2-phenylindole) was used to visualize cell nuclei (blue fluorescence in all panels). Confocal imaging. Scale bar corresponds to 25 μm

Tissue-based indirect immunofluorescence assays (TBA-IFA) using unfixed, frozen rat cerebellum and rat hippocampus brain sections as test substrates, respectively, showed granular staining of the molecular layer of the cerebellar cortex with notable parasagittal stripes and strong membranous staining of a subgroup of hippocampal neurons located at the border between the hilus and the hippocampal granular layer (Fig. 2). IIF employing cultured mouse Purkinje cells (PC) showed membrane-accentuated staining of the PC dendritic tree and soma, which was detected in fixed and permeabilized PC but not in live-cell assays (Fig. 3).

#### Exclusion of previously known anti-neural antibodies

Recombinant cell-based immunofluorescence assays (RC-IFA) covering a panel of 35 established neuronal autoantigens (Euroimmun, Lübeck, Germany) yielded negative results for the sera of all patients (see the *Methods* section for a detailed panel description).

#### Identification of copine-5 as the neuronal target antigen

**Fig. 4:**
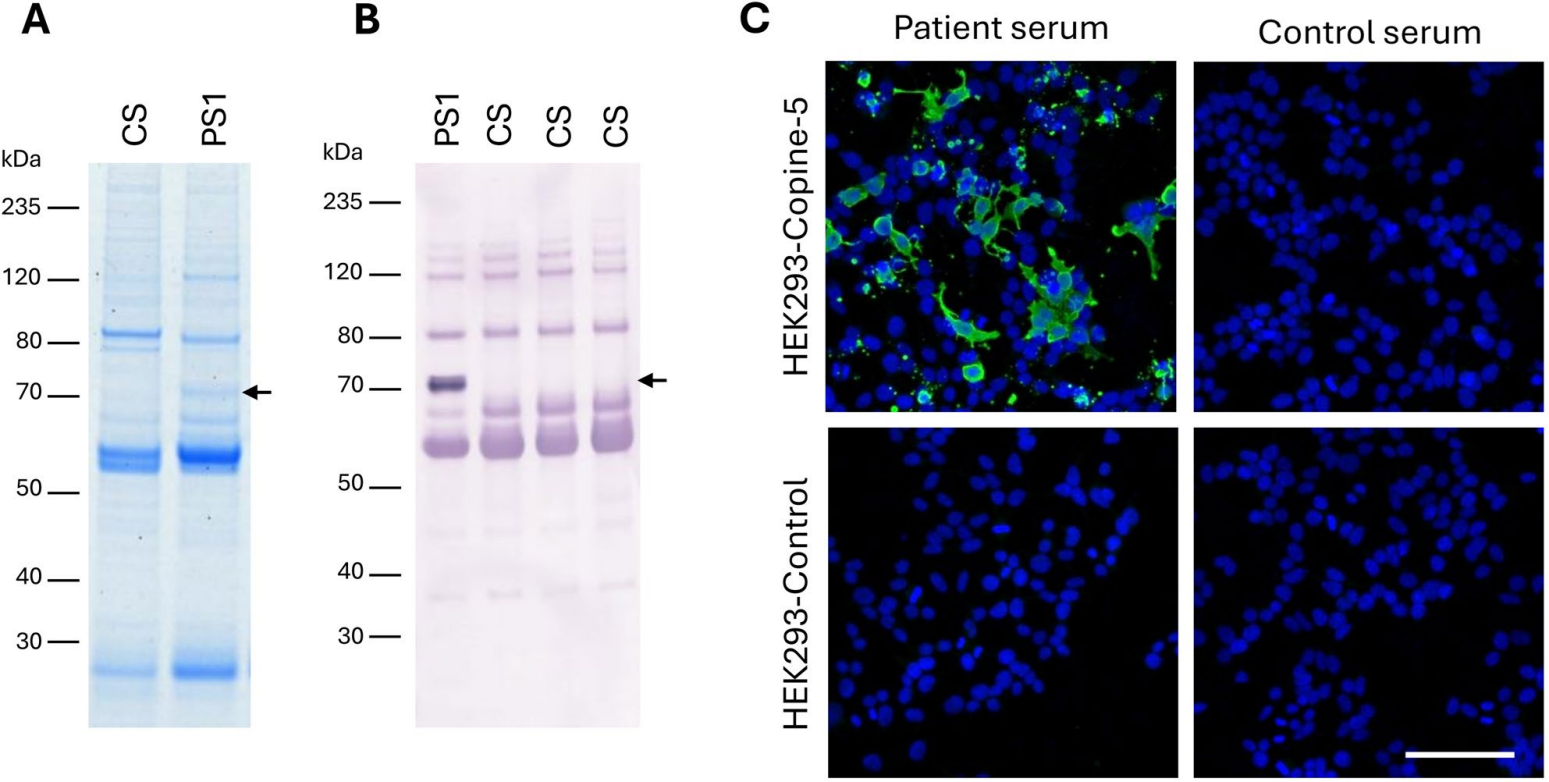
Identification of copine-5 as target antigen. **A** Immunoprecipitation using lysate of rat hippocampus as substrate and serum from patient 1 (PS1) revealed a band at approximately 70 kDa not seen with healthy control serum (CS). Matrix-assisted laser desorption/ionization – time-of-flight (MALDI-TOF) subsequently demonstrated copine-5 as the antigen. **B** In accordance with this finding, a 70-kDa band was observed with a commercial anti-copine-5 antibody in immunoprecipitations from rat hippocampus using serum from patient 1 (PS1) but not using serum from three healthy controls (CS). **C** Binding of serum IgG from patient 1 (left upper panel) but not from a healthy donor (right upper panel) to HEK293 cells transfected with full-length human copine-5. Empty vector-transfected HEK293 cells served as controls (lower panels). Binding of patient IgG was visualized by use of a FITC-labelled, Fcγ-specific goat anti-human IgG secondary antibody (green). TO-PRO-3 stain was used to stain nuclei (blue). Scale bar: 100 μm

Serum from patient 1 was subjected to IP using lysate of rat hippocampus as substrate. SDS-PAGE with the eluate fraction showed a band at approximately 70 kDa which was not present in the control fraction (Fig. 4A; Supplementary Fig. 1). Mass spectrometric analysis of that band revealed copine-5 as the target antigen. IP followed by western blotting with a commercial anti-copine-5 antibody (Sigma Aldrich HPA031369) confirmed pulldown of copine-5 from rat hippocampal lysate for patient serum 1 but not for the controls (Fig. 4B; Supplementary Fig. 2).

#### Detection of anti-copine-5 autoantibodies by recombinant IIFA

Binding of serum IgG from patients 1–4 but not from 50 HD to HEK293 cells transfected with full-length human copine-5 in a cell-based IIFA confirmed correct identification of the target antigen (Fig. 4C). Titers ranged between 1:1000 and 1:100,000 in the serum (individual values: 1:32,000; 1:100,000; 1:1000; 1:32,000) and between 1:320 and 1:10,000 in the CSF (1:10,000; 1:320; not determined in two patients) (Table 1).

#### Competitive immunofluorescence assay

**Fig. 5:**
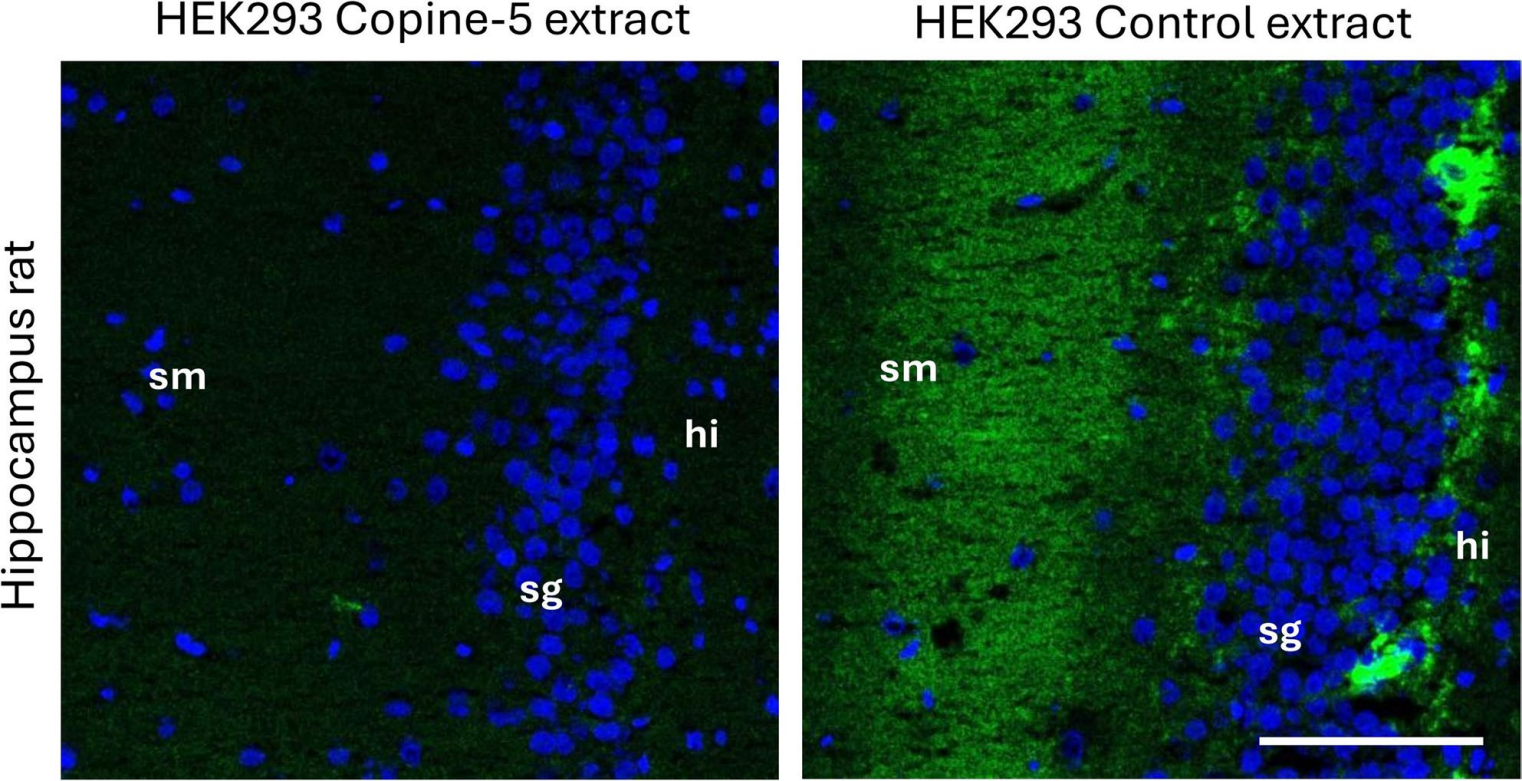
Competitive tissue immunofluorescence results. Preincubation of the index patient’s serum with HEK293 cell extract containing overexpressed copine-5 resulted in complete loss of binding to hippocampus tissue (left panel), while preincubation with cell extract from non-transfected HEK293 cells did not (right panel). Bound patient IgG was visualized by using an Alexa Fluor^®^ 488-labeled, Fcγ-specific goat anti-human IgG secondary antibody (green). Blue coloration corresponds to TO-PRO-3 used to stain cell nuclei. Scale bar: 100 μm

To confirm that the observed tissue IIFA pattern was associated with anti-copine-5 IgG, a competitive IIFA was performed. To that end, a serum sample from patient 1 was preincubated with HEK293 cell extract containing overexpressed copine-5 or with a cell extract from non-transfected HEK293 cells prior to incubation of rat hippocampus sections. While preincubation with the control cell extract did not have any effect on the observed immunofluorescence pattern, preincubation with the copine-5-containing extract completely abolished autoantibody binding (Fig. 5).

#### IgG subclass analysis

Serum and CSF copine-5-IgG belonged predominantly to the strongly complement-activating IgG1 subclass when tested in the TBA-IIFA (patient 2; not tested in the remaining patients); no significant staining of cerebellum or hippocampus tissue sections was seen using secondary antibodies specific for IgG2, IgG3, or IgG4 (not shown). Using the copine-5-specific CBA, IgG1 was the predominant subclass in all four patients (no. 1: IgG1 > > IgG2; no. 2: IgG1; no. 3: IgG1 > > IgG3; no. 4: IgG1 > IgG3 > IgG2).

#### Assessment of intrathecal copine-5-IgG synthesis

CSF/serum ratios for total IgG (QIgG) and CSF/serum ratios for albumin (QAlb) were available from patient 2 (see above for exact values). Based on QIgG, QAlb, a serum copine-5-IgG CBA titer of 1:100,000, and a CSF titer of 1:10,000, an AI of 6.99 (cut-off 4) was calculated, indicating intrathecal synthesis of copine-5-IgG. The same patient also showed strong total IgG synthesis, as evidenced by an elevated QIgG and an intrathecal total IgG fraction of 25%. In patient 3, the AI could not be formally calculated, since QIgG and QAlb were not readily available. However, based on a CSF/serum ratio for total IgG of 1/100–1/2000 in healthy individuals, a CSF/serum ratio of copine-5-IgG CBA titers of ~ 1/3 (1:320/1:1000) suggests possible intrathecal synthesis of copine-5-IgG also in this patient. Copine-5-IgG CSF titers were not assessed in patient 1 and no stored CSF was available for retrospective testing.

## Discussion

We report herein the detection of novel autoantibodies targeting copine-5 in patients with autoimmune encephalitis (AIE) and melanoma. Clinically, three of four patients (no data were available for the fourth patient) developed subacute neuropsychiatric symptoms including memory impairment, confusion and disorientation, psychosis, apathy, altered consciousness, and generalized seizures. In addition, frequent clinical and/or radiological involvement of the basal ganglia (BG), sites of high copine-5 expression [10, 11], was noted. Symptoms in patients with BG involvement included movement disorders and abulia/hypokinetic mutism/psychic akinesia. BG inflammation extended to the internal capsule in two patients, and was associated with paresis of the right leg in one of them. Further symptoms included sixth nerve palsy, dysarthria and/or dysphagia, and painful tonic spasms. AIE can be diagnosed with reasonable certainty with and without the detection of autoantibodies [4]. Autoantibody-associated AIE frequently involves anti-neural antibodies against cell-surface antigens such as glutamate receptors of the NMDA or AMPA type, GABA_B receptors, or LGI1. Other patients present with autoantibodies against intracellular antigens, including ‘classical’ onconeuronal antibodies such as anti-Hu, anti-Ri, anti-Yo, or anti-Ma2; these patients are thought to be less responsive to immunotherapy [4, 12]. Tumors are found at variable rates, such as lung, breast, thymus, or ovarian cancers. In patients with paraneoplastic AIE, treatment of the associated tumor remains the mainstay of therapy. All established, as well as many rare, autoantibodies previously reported in association with AIE were excluded in our patients during the diagnostic workup, along with other, non-autoimmune causes of encephalitis.

Paraneoplastic neurological syndromes (PNS) are uncommon in patients with melanoma, in particular in patients treated with checkpoint inhibitors, and limbic encephalitis has been described in only very few cases [13, 14]. In one patient only, AMPA receptor autoantibodies were detected [15]. Other PNS reported in patients with melanoma include, among others, cerebellar degeneration, opsoclonus-myoclonus syndrome, necrotic myelopathy, dermatomyositis, and demyelinating polyneuropathy [16]. The best-characterized autoantibody reactivity related to melanoma-associated PNS is anti-TRPM1 (transient receptor potential melastatin 1) in melanoma-associated retinopathy [17], while other paraneoplastic neuronal antibodies may occur but are less specifically linked to melanoma [13, 18]. It is therefore of particular note that all four of our copine-5-IgG-positive patients had histologically confirmed melanoma. Other tumor associations may emerge in the future, but the fact that the cases reported here were identified during routine clinical testing of unselected patients suggests a potentially strong association with melanoma.

The frequency of copine-5-IgG-related AIE among all AIE cases is currently unknown. The four copine-5-IgG-positive patients reported here were identified between 2017 and 2024 based on a characteristic binding pattern observed in the IIF-TBA. It is a limitation, however, that the copine-5-specific cell-based assay (CBA) – which yielded higher titers than the TBA and may therefore be more sensitive – was only developed in 2019. Furthermore, the CBA was used only when a TBA-IIF signal suggestive of copine-5-IgG positivity was observed, and it is not known whether all copine-5-IgG-positive patients also show reactivity in the TBA. A significantly lower sensitivity of TBA than recombinant CBA has been reported in several well-established autoantibody-associated autoimmune disorders of the central nervous system, such as aquaporin-4 (AQP4)-IgG-positive neuromyelitis optica spectrum disorders [19] and, in particular, myelin oligodendrocyte glycoprotein (MOG)-IgG-associated disease [20]. The actual frequency of copine-5 autoimmunity may therefore be higher than estimated based on the current data.

Importantly, the onset of AIE preceded the diagnosis of melanoma in three of four patients, and the primary melanoma remained occult in all of them, with no clinically apparent cutaneous, retinal or oral mucosal melanoma present at last follow-up. Whole-body tumor screening by CT or PET-CT may thus be of particular importance in copine-5-IgG-positive patients with PNS.

Before copine-5-IgG was identified, nivolumab-induced immune encephalitis was suspected in patient 3. Melanoma therapy with PD-1 receptor inhibitors/immune checkpoint inhibitors may indeed cause secondary autoimmunity, including PNS, and a broad range of associated antibodies has been reported. However, the antibodies observed in these patients are not melanoma-specific autoantibodies per se [13]. Whether the occurrence of copine-5-IgG-positive AIE in our patient was related to treatment with nivolumab or developed independently of that treatment is unknown. Studies on the frequency of copine-5-IgG among patients with AIE associated with treatment with PD-1 inhibitors or other immune checkpoint inhibitors are warranted.

Copines are soluble cytoplasmic phospholipid-binding proteins conserved from unicellular organisms (e.g., Paramecium) to humans. Copine-5 is highly expressed in the brain, especially in retinal horizontal cells, oligodendrocyte precursor cells, and excitatory neurons and is thought to function as an effector in Pbx (pre-B-cell leukemia homeobox transcription factors)-dependent pathways that influence neuronal differentiation, synaptic connectivity, and organization [21].

Interestingly, brain copine-5 mRNA expression levels are highest in the BG, according to data from the Human Protein Atlas (HPA) project [10], with the strongest copine-5 expression (out of 61 anatomical sites studied, including 21 brain regions) found in the caudate nucleus [11]. Consistent with this finding, at least two of our copine-5-IgG-positive patients (nos. 2 and 4) developed a movement disorder, which was associated with (extensive) BG lesions, including in the caudate nucleus, in at least one of them (MRI data were not available for the second patient, no. 4). In two patients (nos. 1 and 3), BG lesions were documented in the medical records, but no movement disorder; as a limitation, only a basic neurological examination was performed in one of them (patient 3), due to hypoactive delirium, which made it impossible to formally rule out a movement disorder in this case. BG lesions can cause not only classical movement disorders but also a state of disturbed motivation and initiation, leading to reduced spontaneous activity. In fact, severe abulia/inertia (resulting in indifference to hunger and a weight loss of 7 kg within 1 week in one case), flat affect, and apathy, progressing rapidly to full-blown psychic akinesia/hypokinetic mutism, occurred in all three patients with available data, possibly due to interruption of the “motivational circuit” linking the prefrontal cortex (especially anterior cingulate and dorsolateral areas) to the BG (caudate nucleus, putamen, globus pallidus) and then to the mediodorsal thalamus [22]. In patients 1 and 2, hypoactive delirium and hypokinetic mutism, respectively, were clearly associated with lesions in the caudate nucleus (Fig. 1), and lenticulostriate lesions were reported in patient 3 (no data available on patient 4). BG involvement may thus be a core feature of copine-5-IgG-associated AIE, although larger cohorts are certainly needed before definitive conclusions can be drawn.

The distinct BG MRI findings observed in our patients may be of particular clinical relevance, as they may prompt testing for copine-5 autoantibodies in patients with suspected autoimmune encephalitis (AIE). However, further studies are needed to define the full spectrum of MRI abnormalities associated with copine-5-IgG-related autoimmunity.

Whether the dysarthria and dysphagia present in at least two patients reflected BG or internal capsule involvement or were caused by brainstem encephalitis is unclear. However, the finding of sixth nerve palsy in patient 1 and possible Gd enhancement in patient 3 are at least compatible with brainstem involvement.

Basal ganglia involvement is relatively rare in paraneoplastic neurological syndromes but has been reported in CV2/CRMP5 autoimmunity and Ma2/Ta encephalitis as well as, occasionally, in anti-Hu (ANNA-1) syndrome, anti-Ri (ANNA-2) syndrome, anti-recoverin syndrome, and anti-GAD65 autoimmunity [23–30]. Extrapyramidal symptoms have also been observed in anti-IgLON5 disease and NMDAR encephalitis [31, 32]. Apathy and akinetic behavior, mostly due to lesions in the anterior cingulate cortex, BG (especially caudate nucleus), or dorsal thalamus, have also been noted in patients with PNS such as CV2/CRMP5 encephalitis, anti-Hu syndrome, and anti-KLHL11 syndrome. BG involvement also occurs in non-paraneoplastic AIE such as anti-dopamine-2 receptor syndrome [33]. In the differential diagnosis, affective disturbances due to mesial temporal lobe involvement, such as in NMDAR encephalitis, anti-GABA_B receptor or anti-LGI1 encephalitis, may also affect motivation and initiation, and a small number of PNS, such as Ma2/Ta encephalitis, may cause hypersomnia.

Despite the abundance of copine-5 in the brain, little is known about its involvement in pathological conditions. No genetic disorders have been described so far, but studies in knockout mice indicate that copine-5 may regulate anxiety, as its absence leads to decreased anxiety-like behavior. Furthermore, genetic variations in copine-5 have been linked with alcohol dependence and obesity, suggesting its involvement in neuropsychiatric disorders in humans [34].

As is the case for most paraneoplastic neurological syndromes, and given that copine-5 is an intracellularly expressed antigen that was not recognized by patient IgG in the live-cell assay used in our study, the antibody may represent a diagnostically useful biomarker rather than a direct mediator of pathogenesis. Instead, the inflammatory process might be driven predominantly by T cells. Further studies, including passive transfer experiments, are needed to address this question definitively.

Whether copine-5-IgG may be useful as a tumor marker in patients with suspected melanoma and whether copine-5 could serve as a diagnostic histopathological marker for melanoma sub-characterization is currently unknown but is the subject of ongoing studies at the authors’ institutions. While melanoma biopsies were not available for analysis in this study, assessing ectopic copine-5 expression in tumor tissue will be important in future studies to corroborate the notion of a paraneoplastic etiology of AIE in copine-5-IgG-positive patients. According to data from *The Cancer Genome Atlas* (TCGA) project, copine-5 is indeed enriched in skin cutaneous melanoma at the RNA level [35]. The *Human Protein Atlas* project found high or moderate copine-5 expression (mainly cytoplasmic and membranous) in 7/11 patients with melanoma of the skin [10]. Immunohistochemical studies have demonstrated copine-5 expression both in primary melanoma and in melanoma metastases [10], with significant differences in expression levels among patients (Supplementary Fig. 3).

## Conclusion

Copine-5 is a novel autoantibody target in AIE in patients with melanoma. Clinically, copine-5-IgG autoimmunity is characterized by neuropsychiatric and brainstem symptoms, and, of particular note, involvement of the BG. Testing for anti-copine-5 antibodies should be included in the diagnostic work-up of patients with AIE, especially, but not exclusively, if associated with suspected or confirmed melanoma, positive melanoma-associated tumor markers, or lymphadenopathy compatible with metastasis from an unknown primary. Further studies investigating the immunopathogenesis of copine-5-related AIE as well as the potential significance of anti-copine-5 as a novel serological marker and of copine-5 as a potential histopathological tumor marker in patients with suspected melanoma are warranted.

## Supplementary Information

Supplementary Material 1.

## Data Availability

All data supporting the findings of this study are available within the paper.

## Appendix

### Case reports

#### Patient 1

This 64-year-old previously independent woman presented with double vision and reported a 1-week history of fatigue, loss of appetite, and weight loss. On examination, she appeared slightly lethargic. She had a medical history of arterial hypertension, obesity, hypothyroidism, osteoarthritis, carpal tunnel syndrome, and sleep apnea. Her surgical history included hysterectomy, unilateral adnexectomy, and various orthopedic operations. Except for sixth nerve palsy, the neurological examination was normal, as was the initial MRI of the brain. However, CSF analysis showed marginal lymphocytic pleocytosis (6 cells/µl), prompting antibiotic and antiviral treatments. After laboratory tests ruled out bacterial and viral infections, AIE was suspected. Three months after symptom onset, testing for anti-neural antibodies by indirect immunofluorescence showed staining of the membrane of a few rat hippocampal neurons (serum 1:1000 +++) and fine granular staining of the molecular layer of rat cerebellar sections with emphasis around the Purkinje cells (serum 1:320 +++) while testing for 35 different neuronal antibodies by CBA revealed negative results (Laboratory Stöcker, Groß Grönau, Germany).

Treatment was switched to prednisone (1 mg/kg), which was associated with improvement of her clinical symptoms.

Two weeks after symptom onset, she developed a tonic-clonic seizure, prompting addition of levetiracetam. Follow-up CSF analysis showed normalization of the CSF cell count. However, MRI now displayed multiple T2 FLAIR hyperintense, contrast-enhanced lesions in subcortical, paraventricular, hippocampal, and internal capsular locations (Fig. 1, A-C).

The patient’s clinical condition continued to deteriorate, manifesting as progressive hypoactive delirium, characterized by drowsiness, apathy, depressive symptoms, and cognitive-memory deficits.

Evaluation for a paraneoplastic cause by PET-CT demonstrated FDG uptake in an enlarged axillary lymph node. A lymph node biopsy identified a metastatic pleomorphic tumor, consistent with an amelanotic melanoma (S100 positive, AE1/AE3 and CK7 negative; BRAF-mutation V600E). Comprehensive diagnostic workup, including gynecological and dermatological clinical evaluations, colonoscopy, and bone marrow biopsy, failed to locate a primary tumor or further metastasis. The tumor was thus classified as amelanotic BRAF+ (V600E) melanoma of unknown primary origin. Therapy with vemurafenib and cobimetinib was initiated due to the presence of the BRAF V600E mutation.

High-dose methylprednisolone (1 g/day for 5 days) was administered without significant improvement, prompting plasmapheresis (5 × over 5 days) and induction of immunotherapy with three cycles of cyclophosphamide (first cycle: 15 mg/kg, second and third cycles: 13 mg/kg). The patient subsequently improved considerably and was discharged for rehabilitation therapy.

The initial sample was further processed to identify the target antigen, as described in the methods section. Testing in a newly developed copine-5-specific CBA confirmed specificity for copine-5 (serum: 1:32,000; CSF not available for testing).

In the following months, the patient developed further, non-neurological complications, such as splenic infarction, thrombosis of the abdominal aorta, and repeated urinary tract infections. Approximately 6 months later, she was readmitted to hospital with reduced consciousness, elevated inflammatory markers, and elevated renal retention parameters (systemic inflammatory response syndrome, SIRS). Antibiotic treatment was commenced, but it was agreed that no further intensive care would be initiated. The patient was treated palliatively and died shortly thereafter, approximately 7 months after onset of the first symptoms.

#### Patient 2

A 70-year-old male patient developed weakness of his right leg, gait instability, and subacute cognitive dysfunction clinically manifesting as forgetfulness, memory dysfunction, personality changes, lethargy, and almost no spontaneous speech production. Neurological and neuropsychological examination performed two months later showed mild dysarthria, hypomimia, and rigidity of the right extremities, postural instability, tremor of the right hand, hypophonia, micrographia, and a distal central paresis of the right leg, as well as mild impairment of verbal and figurative memory, verbal fluency, and mental flexibility. Cranial MRI disclosed bilateral, prominently left-sided T2/FLAIR signal abnormalities extending from the BG to the temporal lobe with minor gadolinium enhancement in T1-weighted sequences (Fig. 1). CSF analysis revealed 8 white cells/µl with few plasma cells on cytologic assessment, an elevated CSF/serum albumin ratio (QAlb = 13.1), reflecting moderate blood/CSF barrier dysfunction, and intrathecal IgG synthesis (elevated QIgG [14.3]; intrathecal Ig fraction 25.3%; normal QIgA and QIgM). CSF L-lactate levels were normal. Screening for infections was negative (PCR for HSV-1-, HSV-2-, and varicella-zoster-virus DNA; serology for *Borrelia burgdorferi*). Testing for anti-neural antibodies three months after symptom onset by indirect immunofluorescence showed staining of the membrane of a few rat hippocampal neurons and fine granular staining of the molecular layer of rat cerebellar sections (CSF 1:100 +++; serum 1:320 +++) (Laboratory Stöcker, Groß Grönau, Germany). A broad panel of monospecific tests was negative (see Table for details), suggesting the presence of an as yet unknown anti-neuronal antibody. Five months after neurological symptom onset, testing of a second paired CSF/serum sample on rat cerebellum and rat hippocampal tissue sections at the University of Heidelberg showed prominent staining of the cerebellar molecular layer with notable parasagittal stripes, processes in contact with the Purkinje cell membrane, and moderate staining of the granular cells of the cerebellum (serum > 1:1000, CSF > 1:50 and < 1:500), along with staining of a subset of hippocampal neurons (serum > 1:1000, CSF > 1:50 and < 1:500) using an Fc-specific goat anti-human IgG detection antibody (Euroimmun, Lübeck, Germany). No serum or CSF IgM or IgA binding was seen either on cerebellar or hippocampal sections. No distinct staining of primate peripheral nerve sections was seen. Brain MRI still showed frontal-predominant periventricular T2/FLAIR hyperintensities; native T1 hyperintensities and susceptibility weighted imaging (SWI) voids in the BG; SWI voids in the anterior commissure; brain atrophy, mainly due to predominantly left-sided basal ganglia atrophy (mostly affecting the head of the caudate nucleus) and atrophy of the left hippocampus/mesial temporal lobe, dilatation of the temporal horn of the lateral ventricle, and widening of the external CSF spaces. Since clinical, imaging, CSF, and serological findings suggested an autoimmune and potentially paraneoplastic origin of the patient's symptoms, an extended clinical work-up was performed. Thoracic CT scanning revealed a spiculated mass in the left lung apex with broad-based pleural involvement along with mediastinal lymph nodes suspicious for metastases at the aortic arch. A biopsy followed by resection of the pulmonary nodule confirmed malignant melanoma (cTX cNX pM1b stage IV; mutational state: BRAF p.Val600Glu, activating mutation; TERT Promoter C250T, activating mutation; ERBB3 p.Leu97fs*26, deleting mutation). S100 and LDH as laboratory serum markers for progression of metastatic melanoma were normal. A short course of high-dose intravenous methylprednisolone (1000 mg daily for 3 days) resulted in transient but not sustained amelioration of clinical symptoms. Oncological treatment with the oral BRAF/MEK inhibitors encorafenib and binimetinib, commenced at month 5, was paralleled by stable findings in CT scans of neck, lung, abdomen, and pelvis performed at 3-monthly intervals, but had to be discontinued seven months later due to dermatological side effects. At the same time, the patient experienced progressive neurological deterioration including painful tonic spasms of the right extremities refractory to treatment with gabapentin, episodes of stabbing pains in the right groin and leg, and focal allodynia in the right lower abdomen requiring combined therapy with pregabalin and amitriptyline, as well as marked hypophonia and increasing impairment of verbal memory and verbal fluency. Following a severe COVID-19 infection at month 11 he remained nearly permanently bedridden. At last follow-up, markedly reduced motivation and initiation as well as hypokinetic mutism were present. Follow-up MRI 12 months after onset still showed bilateral, frontal-predominant T2/FLAIR lesions. Signal abnormalities in the BG and temporal lobe may have declined to some extent in a follow-up cranial MRI at month 15 (although T2/FLAIR sequences were not performed), but dilatation of the lateral ventricles was noted. Repeated CSF assessments (months 5 and month 16) revealed elevated QAlb (18.5; 10.7) and CSF-restricted oligoclonal bands (1 x type 2; 1 x type 3) but normalized QIgG and CSF white cell count. At month 16, follow-up immunofluorescence testing for anti-neural antibodies still showed membranous staining of a few hippocampal ganglion cells and a fine speckled immunofluorescence pattern of the cerebellar and hippocampal molecular layer. Retrospective testing of the initial sample in a newly developed CBA confirmed specificity for copine-5 (serum: 1:100,000, CSF: 1:10,000).

#### Patient 3

This 75-year-old man had a medical history of arterial hypertension, diabetes mellitus, hyperlipidemia, peripheral artery disease, venous insufficiency, and benign prostatic hyperplasia. Nine months before the onset of neuropsychiatric symptoms, he had been diagnosed with a metastatic melanoma (pT3b, N3c, cM1b; AJCC stage IV). Treatment included tumor excision, radiation therapy, and immunotherapy with nivolumab and ipilimumab (BRAF-wt). The initial neurological symptoms leading to admission were dysarthria and dysphagia. Cranial MRI changes reportedly included a lesion in the left putamen, lenticulostriate and insular lesions, hippocampal changes considered to correspond to remnant cysts, and possible gadolinium enhancement in the medulla oblongata and pons (data not shown). In addition, global brain atrophy with expansion of the inner and outer CSF spaces, both supra- and infratentorially, and marked periventricular white matter T2 hyperintensities were noted. However, a microangiopathic origin of some of the lesions was considered in the differential diagnosis by the interpreting radiologist. CSF analysis revealed mild pleocytosis (24 cells/µL) and elevated protein levels (106 mg/dL). Testing for anti-neural antibodies by indirect immunofluorescence showed staining of the membrane of a subset of rat hippocampal neurons and fine granular staining of the molecular layer of rat cerebellar sections while testing for neuronal antibodies by CBA and line blot revealed negative results (Laboratory Krone, Bad Salzuflen, Germany). Laboratory testing was otherwise unremarkable, leading to suspicion of nivolumab-induced immune encephalitis. Corticosteroid treatment was initiated with some effect, prompting a 3-day course of IVIG, after which the patient’s symptoms improved and he was discharged.

Four weeks later, the patient was re-admitted due to somnolence, affective incontinence, and fluctuating confusion and aggression. Dysarthria and dysphagia had progressed. Repeat cranial MRI showed no acute lesions except for areas of suspected leukoencephalopathy. CSF analysis showed mild pleocytosis (7 cells/µl), blood-CSF barrier dysfunction, CSF-specific oligoclonal IgG bands, but no quantitative evidence of intrathecal IgG, IgA, or IgM synthesis (normal QIgG, QIgM, and QIgA), and a slight increase in CSF protein (46 mg/dl). No morphological or immunohistochemical evidence of malignant cells in the CSF was found. Autoantibody panel testing was negative. Glucocorticoid therapy was repeated at 1 g/d for 5 days, with no significant clinical effect. At last follow-up, the patient was cared for in a nursing home. Retrospective testing of the initial sample in a newly developed CBA confirmed specificity for copine-5 (serum: 1:1000, CSF: 1:320).

## Abbreviations

AI: Antibody index
AIE: Autoimmune encephalitis
AJCC: American Joint Committee on Cancer
AMPAR: α-amino-3-hydroxy-5-methyl-4-isoxazolepropionic acid receptor
ANNA-3: Anti-neuronal nuclear antibody type 3
AP3B2: Adaptor-related protein complex 3, beta 2 subunit
AQP4: Aquaporin-4
ARHGAP26: Rho GTPase-activating protein 26 (anti-Ca)
ATP1A3: ATPase Na+/K+ transporting subunit alpha 3
BB: Borrelia burgdorferi
BCB: Blood-CSF barrier
BG: Basal ganglia
BRAF: v-Raf murine sarcoma viral oncogene homolog B1
BSA: Bovine serum albumin
BSTE: Brainstem encephalitis
CBA: Cell-based assay
CK7: Cytokeratin 7
CPNE5: Copine-5
CSF: Cerebrospinal fluid
CT: Computed tomography
CYP: Cyclophosphamide
DAPI: 4´,6-diamidino-2-phenylindole
DNER: Delta/Notch-like EGF-related receptor
DPPX: Dipeptidyl-peptidase-like protein 6
EEG: Electroencephalogram
ERBB3: Receptor tyrosine-protein kinase erbB-3
FLAIR: Fluid-attenuated inversion recovery
TBE: Tick-borne encephalitis
GABA_A-R: Gamma-aminobutyric acid receptor type A
GABA_B-R: Gamma-aminobutyric acid receptor type B
GAD65: Glutamic acid decarboxylase 65
Gd: Gadolinium
GluR-delta2: Glutamate receptor delta-2 subunit
GlyR: Glycine receptor
HSV1/2: Herpes simplex virus types 1 and 2
IgG: Immunoglobulin G
IgG-IF: Intrathecal IgG fraction
IgLON5: Immunoglobulin-like cell adhesion molecule 5
IM/IS: Immunomodulatory/immunosuppressive
IP: Immunoprecipitation
ITPR1: Inositol 1,4,5-trisphosphate receptor type 1 (anti-Sj)
IVIG: Intravenous immunoglobulin
IVMP: Intravenous methylprednisolone
KCNA2: Potassium voltage-gated channel subfamily A member 2
LGI1: Leucine-rich glioma-inactivated 1
LN: Lymph node
LP: Lumbar puncture
ly-mo: Lympho-monocytic
Ma1: Ma1 onconeural antigen
Ma2: Ma2 onconeural antigen
MEK: Mitogen-activated protein kinase kinase
mGluR1: Metabotropic glutamate receptor 1
MALDI-TOF: Matrix-assisted laser desorption/ionization – time-of-flight
mGluR5: Metabotropic glutamate receptor 5
mRS: Modified Rankin Scale
MRI: Magnetic resonance imaging
MO: Medulla oblongata
MOG: Myelin oligodendrocyte glycoprotein
MRZ: Measles, rubella, and zoster reaction
NF155: Neurofascin 155
NF186: Neurofascin 186
NGS: Normal goat serum
NMDAR: N-methyl-D-aspartate receptor
OCB: Oligoclonal IgG bands
PBS: Phosphate-buffered saline
Pbx: Pre-B-cell leukemia homeobox transcription factors
PD-1: programmed death 1
PET: Positron emission tomography
PEX: Plasma exchange
PNS: Paraneoplastic neurological syndrome
QAlb: Albumin CSF/serum ratio
QIgG: IgG CSF/serum ratio
Ri: Ri onconeural antibody
SEZ6L2: Seizure-related 6 homolog-like 2 protein
sg: Stratum granulosum
SIRS: Systemic inflammatory response syndrome
sm: Stratum moleculare
SOX-10: SRY-related HMG-box gene 10
Ta: Onconeural antigen Ta (same as Ma2)
TERT: Telomerase reverse transcriptase
TNM: Tumor node metastasis staging system
TP: Total protein
VZV: Varicella-zoster virus
WCC: White cell count
WM: White matter
wt: Wild type
Yo: Yo onconeural antibody
Zic4: Zinc finger protein 4
