# Supplementary material for: Copine-5-IgG-related autoimmune encephalitis: a novel paraneoplastic neurological syndrome with a strong melanoma association
Source: J Neuroinflammation. 2026 Jul 27;23:258. doi: 10.1186/s12974-026-03793-4 (PMC13411103; doi:10.1186/s12974-026-03793-4)
Supplement: Supplementary file 1 — Supplementary Material 1. [file 12974_2026_3793_MOESM1_ESM.pdf]

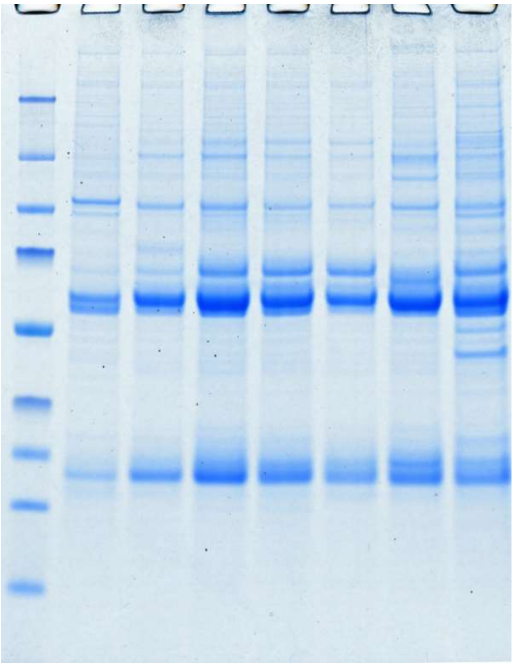

Supplementary Figure 1. Uncropped image for Figure 4A. Immunoprecipitation using rat hippocampal lysate and patient sera and SDS-PAGE stained with Coomassie staining. Lane 1: protein molecular weight marker; lane 2: healthy control serum; lane 3: serum from patient 1; lanes 4–8: sera from other patients with suspected autoimmune disease (not showing an autoantibody-binding pattern similar to that of patient 1 in the tissue immunofluorescence assay).

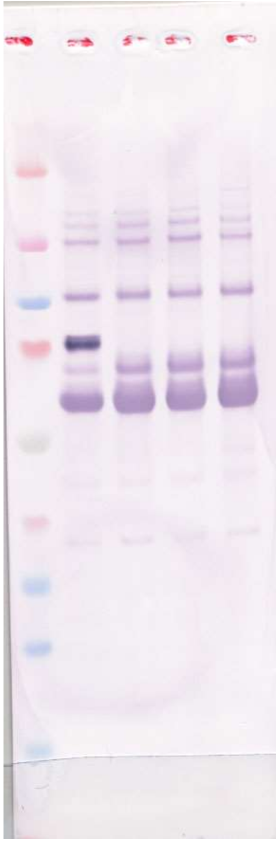

Supplementary Figure 2. Uncropped image for Figure 4B. Immunoprecipitation (IP) using rat hippocampal lysate and patient serum and control sera, followed by Western blotting and incubation with a commercial anti-copine-5 antibody. Lane 1: protein molecular weight marker; lane 2: eluate fraction from IP with serum from patient 1; lanes 3-5: eluate fractions from IP with healthy control sera.

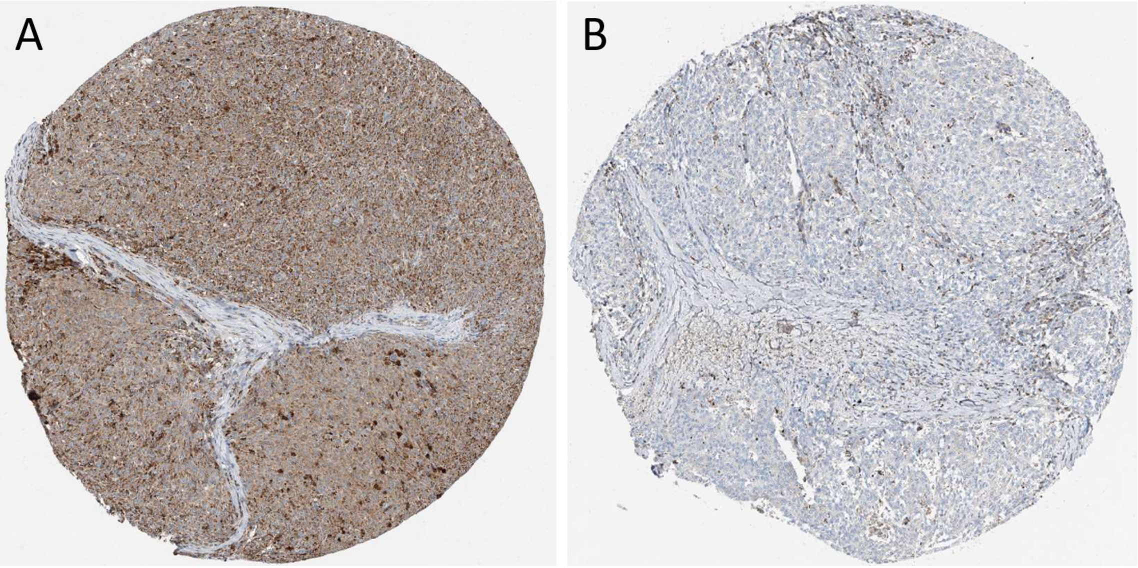

Supplementary Figure 3. Expression levels of copine-5 protein in human melanoma tissue differ between patients. (A) Positive cytoplasmic/membranous staining (moderate intensity) of over 75% of cells in a melanoma (subtype not specified) tissue section obtained from an 82-year-old female patient. (B) Negative staining of a melanoma (subtype not specified) tissue section from a 72-year-old female patient. Copine-5 expression was detected by conventional immunohistochemistry using an affinity-isolated, rabbit polyclonal antibody targeting a 60-aa N-terminal fragment of human copine-5 (MEQPEDMASLSEFDSLAGSIPATKVEITVSCRNLLDKDMFSKSDPLCVMYTQGMENKQW) as primary and a DAB (3,3'-diaminobenzidine)-based detection system. Brown staining indicates antibody binding. Sections are counterstained with hematoxylin to enable visualization of microscopical features. Modified images from the Human Protein Atlas image database (Uhlén M, Fagerberg L, Hallström BM, Lindskog C, Oksvold P, Mardinoglu A, et al. Tissue-based map of the human proteome. Science. 2015;347:1260419) under the terms of the Creative Commons Attribution License (http://creativecommons.org/licenses/by/4.0).
